# Rectal diverticulum in a terrier dog: A case report

**Published:** 2013

**Authors:** Hossein Kazemi Mehrjerdi, Ali Mirshahi, Amir Afkhami

**Affiliations:** 1 *Department of Clinical Sciences, School of Veterinary Medicine, Ferdowsi University of Mashhad, Mashhad, Iran; *; 2 * Department of Pharmacology, School of Veterinary Medicine, Ferdowsi University of Mashhad, Mashhad, Iran.*

**Keywords:** Diverticulum, Dog, Perineal hernia, Rectum

## Abstract

Rectal diverticulum is a rare condition in dogs characterized by formation of a pouch or sac due to hernial protrusion of the mucous membranes through a defect in the muscular coat of the rectum. A 12-year-old male terrier dog was admitted with a history of a left perineal swelling, dyschezia and tenesmus during the last five months. Digital rectal examination identified a weakness in the left pelvic diaphragm and feces-filled sac within the lateral wall of the rectum. Positive contrast radiography showed a marked solitary diverticulum (3.5 × 4 × 4.5 cm) with wide-orifice neck arising from the left rectal wall. Using a lateral approach, a large rectal diverticulum was found and diverticulectomy following standard herniorrhaphy was performed. The dog recovered uneventfully with no signs of dyschezia during the next three years. Diverticulectomy by lateral approach and perineal herniorrhaphy produced excellent results.

## Introduction

Rectal diverticulum is a rare out-pouching of rectal mucous membranes in dogs through a defect in the overlying muscle layers, found mostly in middle aged, male dogs.^1^^,^^2^ The precise cause is still unknown, although it may arise from focal weakened points of the rectal wall due to congenital or acquired causes.^3^ Rectal diverticula may exist alone, but most often it has been described as a sequel to perineal hernia.^4^^-^^7^ It has been known that without efficient repair of the diverticulum, neither conservative treatments nor classical herniorrhaphy techniques would be successful. This problem may lead to incomplete rectal emptying at defecation, persistent straining, and recurrence of hernia. Therefore, surgical correction of rectal diverticula is necessary.^1^^,^^7^ Surgical treatments of this condition include diverticulectomy, plication, anal splitting and resection of the segment of intestine with end-to-end anastomosis. Each technique is associated with morbidity.^1^^,^^7^^-^^9^ This case report describes the successful use of diverticulectomy accompanied by herniorrhaphy in a 12-year-old male dog using a lateral approach.

## Case History

A 12-year-old male terrier dog, weighing 6 kg, was admitted to the Department of Clinical Sciences, Faculty of Veterinary Medicine, Ferdowsi University of Mashhad, Mashhad, Iran; with a five months history of left side perineal swelling, dyschezia and tenesmus. The perineal swelling was warm, soft and painful. The rectal temperature, pulse rate and respiratory rate were within normal ranges. Upon admission, the patient was normal in general condition. The preoperative laboratory evaluation (complete blood count and serum chemistries) was normal. Digital rectal examination revealed a weakness in the left pelvic diaphragm and a large out-pouched pocket located at the left lateral aspect of the rectal wall approximately 2 cm from the anal orifice. 

**Fig. 1:**
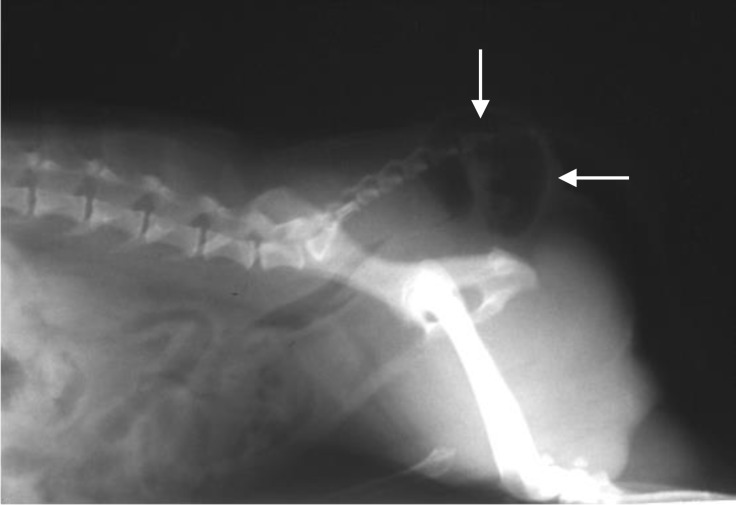
Plain lateral radiograph of caudal abdominal region indicating a gas-filled sac (arrows) in perinea. There are no feces in the diverticulum due to enema performed before radiography.

**Fig. 2:**
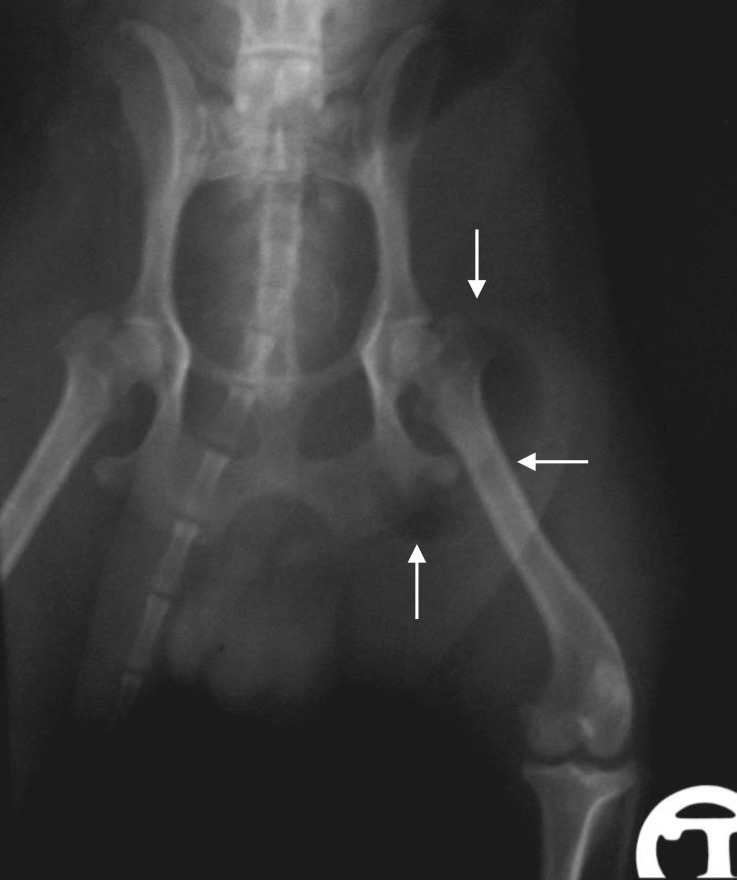
Plain ventrodorsal (VD) radiograph of caudal abdominal region; note that the radiolucent region is superimposed on femoral neck (arrows).

**Fig. 3:**
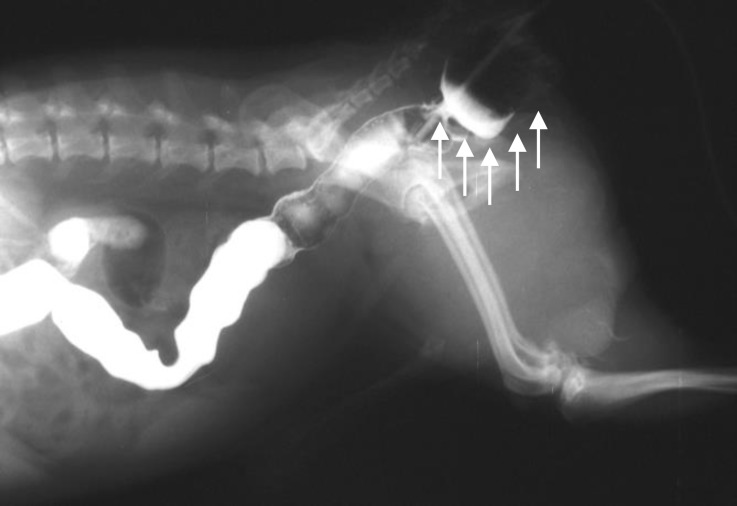
Lateral radiograph following barium sulfate enema and prior to surgery; note that the positive contrast media and Foley catheter trapped (arrows) in the diverticulum.

**Fig. 4:**
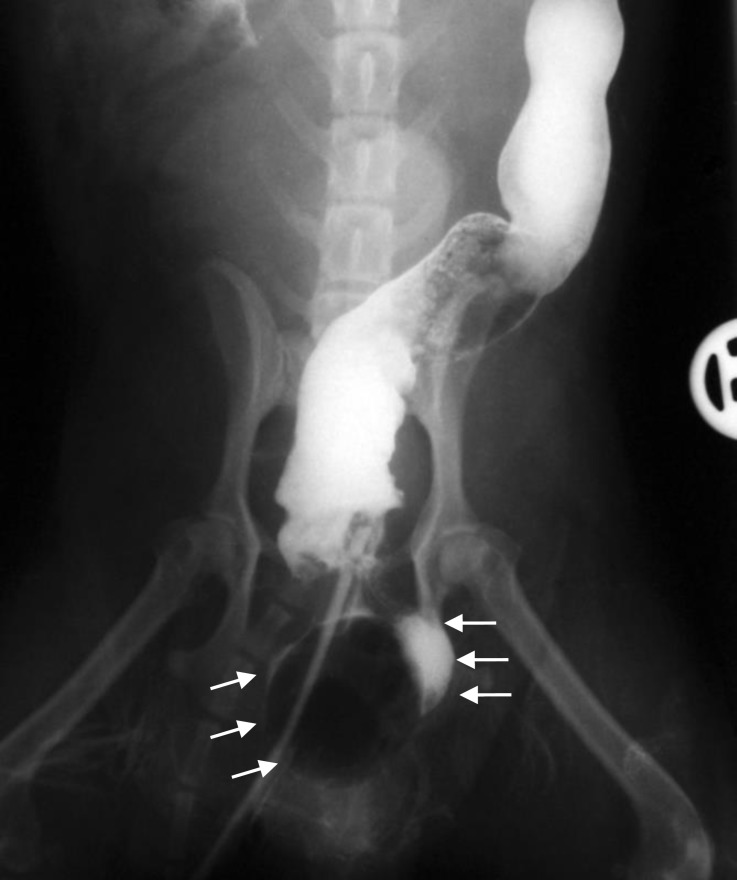
Ventrodorsal radiograph of barium sulfate enema; note that the contrast media and Foley catheter trapped (arrows) in the diverticulum. Rectal diverticulum is outlined by barium sulfate contrast media in the left side

The dog was fed a home prepared diet and housed inside. The owner noticed the perineal swelling five months ago. No history of previous trauma or surgery was reported. Chronic constipation and dyschezia for 6 six years, necessitating rectal evacuations, were also reported by the owner. Digital rectal examination revealed that the left perineal hernia contained a large quantity of feces. In plain radiographs, a gas-filled sac was observed in perineal region with 3.5 × 4 × 4.5 cm dimensions (Figs. 1 and 2). Positive contrast radiography using barium sulfate revealed a marked solitary diverticulum arising from the left lateral wall of the rectum (Figs. 3 and 4).

**Surgery. **After diagnosis of rectal diverticulum and perineal hernia by physical examination and positive contrast radiography, diverticulectomy using lateral approach and herniorrhaphy were scheduled. Feeding was withheld 48 hr before surgery but the dog had free access to water. The patient has experienced a period of mucillium treatment, as laxative, followed by warm water enema for colonic evacuation and cleansing before the surgery. Ceftriaxon (Ceftrax^®^, Jaber Ebne Hayyan Pharmaceutical Co., Tehran, Iran) at the dose of 22 mg kg^-1^, three times daily have been administered intramuscularly before surgery and continued for five subsequent days after surgery.

After premedication with intramuscular acepromazine (0.03 mg kg^-1^, Alfasan, Woerden, The Netherlands), anesthesia was induced with combination of intravenous diazepam (0.2 mg kg^-1^, Caspian Tamin Pharmaceutical Co., Rasht, Iran) and ketamine (6 mg kg^-1^, Alfasan, Woerden, The Netherlands). Anesthesia was maintained with 1.5 to 2% isoflurane (Nicholas Piramal Ltd., London, UK) in 100% oxygen. The dog was positioned in sternal recumbency and fecal material was manually evacuated from rectum. After routine preparation of surgical site, a laterally curved, dorsoventral skin incision was done over the hernia. Diverticulum was identified and a circumferential incision was made through the mucosa and all bulging mucosa was resected. Mucosal simple interrupted-suture pattern was oversewn with a seromascular Cushing pattern using 3-0 polyglactin 910 suture material (Vicryl^®^, Ethicon, Edinburgh, UK) in a parallel fashion to the rectal direction. A second layer of continuous Cushing sutures was placed to reinforce the diverticulectomy closure. The hernia was repaired using conventional technique with 2-0 polydioxanon suture (PDS^®^ΙΙ, Ethicon, Edinburgh, UK). During herniorrhaphy, the first sutures were placed from the internal obturator muscle ventrolaterally to the external anal sphincter medially, and then from the sacrotuberous ligament, the coccygeal muscle and the levator ani muscle laterally to the external anal sphincter medially. Subcutaneous tissues and skin were closed routinely.^8^ Prescrotal castration was done two months after surgery to reduce the risk of recurrence of hernia.

**Fig. 5:**
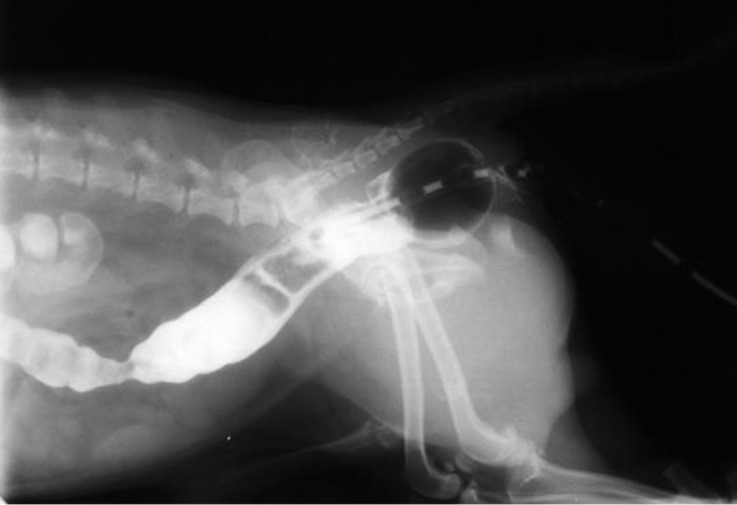
Lateral radiograph of barium sulfate enema two months after surgery; no sign of contrast media accumulation is seen in previous region of rectum

**Fig. 6:**
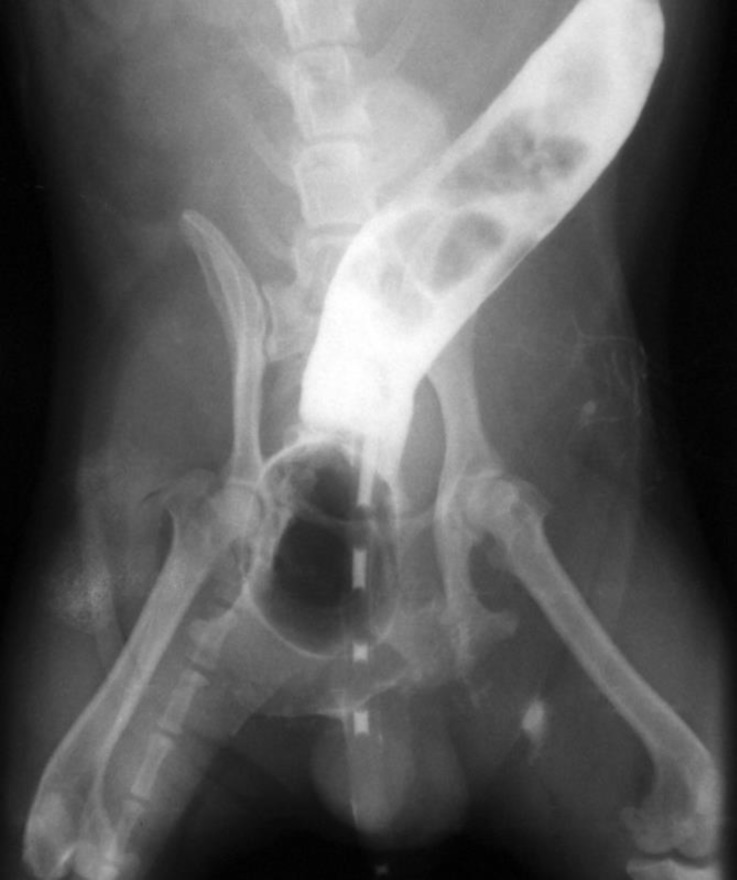
Ventrodorsal radiograph of barium sulfate enema two months after surgery; no sign of contrast media accumulation is seen in previous region of rectum

**Postoperative care and follow-up. **Postoperative treatment included using of an Elizabethan collar, intravenous fluid therapy, and antibiotic therapy. The dog recovered uneventfully and obtained relief from the outlet obstruction. Repeated rectal examination revealed that the wide-orifice neck of the diverticular out-pouching had been tightly closed. The dog was discharged two days after surgery with instructions for warm compresses to relieve perineal swelling. Lactulose was administered as a fecal softener for two weeks. A barium sulfate enema was repeated two months later (Figs. 5 and 6). Three years after surgery, the dog was normal and had no problem related to the perineal hernia. Defecation was normal without the aid of fecal softeners.

## Discussion

Tenesmus and obstipation happen in dogs due to different reasons.^1^ Very rare causes are colorectal diverticula which are well described in the human literature.^10^ Weakness and rupture of muscular layer of the rectal wall and protruding of mucosa and submucosa into the pelvic canal lead to rectal diverticulum.^7^ In humans, colonic diverticula are common, whereas rectal diverticula happen rarely, with an incidence of 0.07-0.08%.^10^^-^^12^ In one study, Hosgood *et al*. investigated rectal abnormalities in 30 dogs. Rectal deviation was recorded in all dogs and rectal sacculation was documented in 12 cases, but no diverticulum diagnosis was reported by radiography or during surgery.^13^

The exact causes of rectal diverticula development are still unknown. In human, some predisposing factors include primary muscular atrophy, congenital defects, increased feces transit time, obesity with fatty infiltration of the rectal wall, relaxed rectal-vaginal septum, pelvic trauma and rectal infections or ulcerations, among them congenital defects seem to represent the major cause.^10^ Recently, rectal diverticula have been reported as surgical complications after the stapled trans-anal rectal resection (STARR) procedure.^12^^,^^14^ In our case, the possible cause of rectal diverticula was recurrent fecal impaction that exerts pressure and causes distension of the rectum. During chronic episodes of constipation, overzealous digital rectal evacuation had weakened the rectal wall with subsequent diverticulum formation.

Most common rectal diseases in dogs such as rectal deviation (or flexure), rectal diverticulum and rectal sacculation occur in combination with perineal hernia. Vnuk *et al*. and Krahwinkel have reported that all dogs enduring rectal diverticula, deviation and sacculation; also suffer from perineal hernia. However, there is no definite understanding about the primary etiology and the exact sequence of theses countercurrent events. However, perineal hernia may occur in dogs independent to other rectal diseases. According to this finding, Krahwinkel concluded that occurrence of rectal disease is a consequence of perineal hernia which develops initially.^7^^,^^8^

In human, most rectal diverticula do not require any treatment procedure, as they are asymptomatic in the majority of patients. Occasionally, they may become inflamed with impacted feces and progress to abscess formation or perforation. Some complications associated with rectal diverticula include diverticulitis, rectal stenosis, fecal impaction in the diverticulum, rectovesical fistula, and rectal prolapse. Surgical intervention is the only option in such complicated patients.^10^^,^^15^^-^^18^

Several surgical techniques can be used to treat rectal diverticulum in dogs.^5^ Conservative treatment or classical herniorrhaphy techniques will not be successful without repairing the diverticulum. Large rectal diverticulum will fill with feces and cause straining, leading to disruption of the perineal hernia repair and recurrence of the perineal swelling.^1^^,^^7^ This may partially account for the high recurrence rates previously reported.^19^^,^^20^ Therefore, surgical correction of rectal diverticulum or sacculation should be carried out to prevent recurrence of perineal hernia. Some surgeons suggest that the solution for this problem is excision of rectal diverticula and large sacculations, followed by suturing the rectal wall at the time of the herniorrhaphy.^1^^,^^7^^,^^8^ During diverticulectomy or sacculectomy, due to the rectal wall opening, the risk of possible contamination can be expected.^8^ Larsen used a method of plication (closed inversion of the rectum by inverting sutures) to reduce the size of rectal diverticula. This technique decreases surgical contamination in an area that is grossly contaminated. If plication is used, rectal prolapse, suture sinuses and seroma formation may occur after the operation.^5^^,^^21^ Anal splitting and resection of the segment of intestine with end-to-end anastomosis have also been described but are challenging and associated with morbidity.^1^^,^^8^^,^^9^ In our presenting case, diverticulectomy using lateral approach and perineal herniorrhaphy produced excellent results. 
